# Tumor heterogeneity as a driver of drug resistance and its implications for personalized therapy

**DOI:** 10.20517/cdr.2025.216

**Published:** 2026-05-12

**Authors:** Erhan Da, Ronglan Zhu, Bin Xi, Liyang Zhang, Guodong Tang

**Affiliations:** ^1^Department of Neurosurgery, Xiangya Hospital, Central South University, Changsha 410008, Hunan, China.; ^2^National Clinical Research Center for Geriatric Disorders, Xiangya Hospital, Central South University, Changsha 410008, Hunan, China.; ^3^Hypothalamic Pituitary Research Center, Xiangya Second Hospital, Central South University, Changsha 410008, Hunan, China.; ^4^Department of Neurosurgery, National Regional Center for Neurological Diseases, Xiangya Hospital (Jiangxi), Central South University, Nanchang 330038, Jiangxi, China.; ^5^Department of Neurosurgery, Jiangxi Provincial People’s Hospital, Clinical College of Nanchang Medical College, First Affiliated Hospital of Nanchang Medical College, Nanchang 330038, Jiangxi, China.

**Keywords:** Tumor heterogeneity, drug resistance, single-cell omics, spatial omics, personalized therapy, targeted therapy

## Abstract

Tumors are highly dynamic diseases characterized by significant heterogeneity. They consist of multiple cellular populations with distinct properties that respond differently to therapeutic pressure. This heterogeneity may arise from spatial variation across tumor regions (spatial heterogeneity) as well as from temporal changes during tumor evolution and treatment (temporal heterogeneity). As a consequence, drug-resistant subclones often emerge under therapy and contribute to treatment failure. Advances in single-cell and spatial multi-omics technologies enable precise quantification of tumor heterogeneity, supporting detailed investigation of how heterogeneity contributes to chemoresistance and informing the development of personalized therapeutic strategies. In this review, we summarize the evolutionary dynamics underlying the emergence of tumor drug resistance and examine the molecular mechanisms responsible for failure of targeted therapies. We highlight how advances in single-cell and spatial multi-omics have significantly improved our ability to elucidate these processes. We further suggest that addressing tumor drug resistance may require a shift from static, single-target approaches toward dynamic, biology-informed personalized strategies. Integrating high-resolution multi-omics monitoring with functional validation could enable identification of subclonal vulnerabilities, support adaptive treatment adjustment, and contribute to more durable clinical responses.

## INTRODUCTION

**Figure 1:**
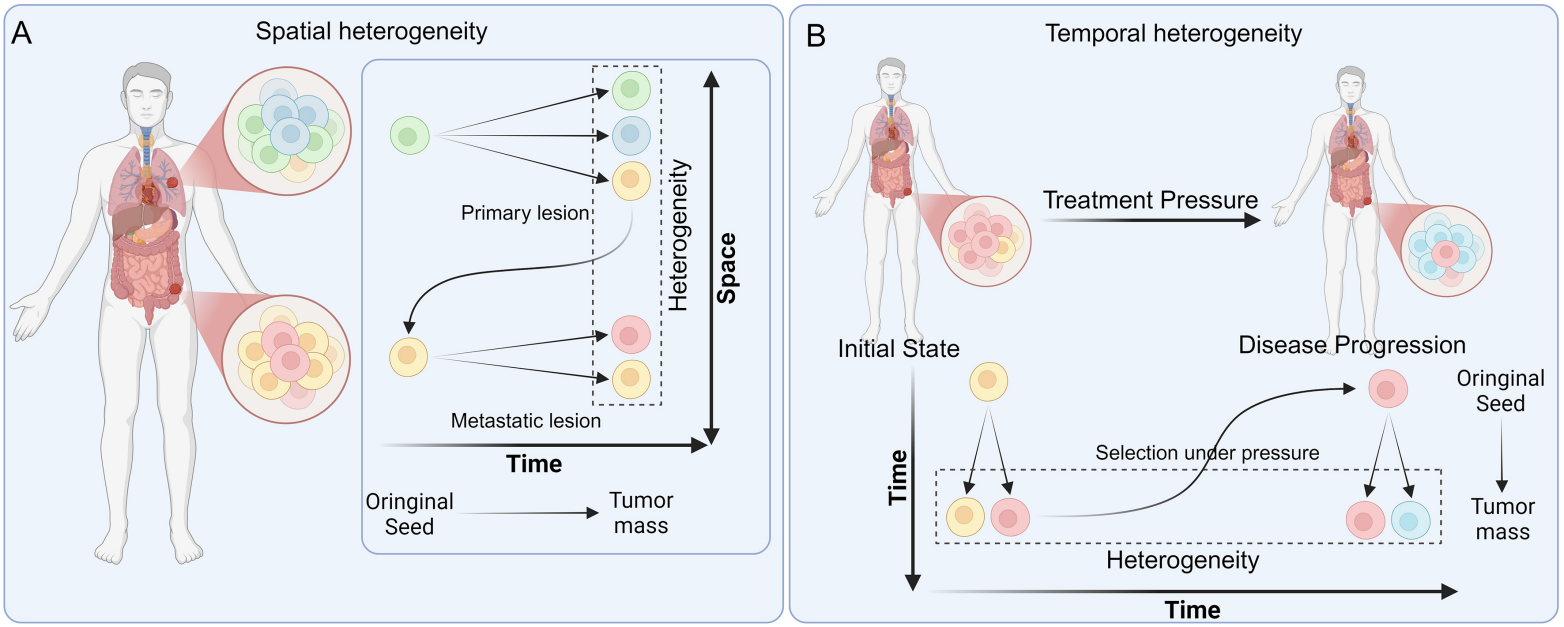
Schematic diagram of spatial and temporal heterogeneity. (A) Spatial heterogeneity. Tumors gradually differentiate into distinct subclones at the primary or at metastatic lesion sites, revealing spatial heterogeneity when sampled and analyzed at a given moment; (B) Temporal heterogeneity. Tumors not only develop distinct subclones during tumorigenesis, but these subclones also undergo progressive selection and evolution under therapeutic pressure. Sampling at different stages of tumor progression reveals temporal heterogeneity. Created in BioRender. Da, E. (2026) https://BioRender.com/8bprxj6.

Tumors represent one of the most prevalent and pressing health challenges globally. They are characterized by extensive molecular, genetic, and phenotypic diversity. Tumor heterogeneity is pervasive, occurring not only across patients but also among different tumors within the same individual and even among cellular populations within a single tumor^[1]^. Tumor development and progression are influenced both by intrinsic genetic alterations and by the microenvironment in which tumor cells reside^[2,3]^. During the transition from normal to malignant cells, a series of genetic and epigenetic alterations arise that confer cells properties such as accelerated proliferation, immune evasion, angiogenesis, invasion, and metastasis^[4-6]^. Since tumor initiation and progression are driven by stochastic events, tumors frequently display remarkable complexity and adaptability^[7]^. Therefore, extensive heterogeneity exists across tumor types, anatomical sites, and individual tumor cells^[1,8]^. Tumor heterogeneity manifests both spatial and temporally and is shaped by intrinsic genetic and epigenetic factors as well as external environmental influences [Figure 1]. Spatial heterogeneity refers to the genetic and epigenetic diversity across different tumor regions, including the tumor microenvironment (TME) in which the tumors reside. Temporal heterogeneity denotes the progressive evolution of the tumor’s genetic, epigenetic, and microenvironmental landscapes throughout disease progression^[1,7]^. This heterogeneity manifests through genetic mutations, epigenetic modifications, and changes in gene expression that ultimately lead to phenotypic diversity. Such diversity has become a major obstacle to effective tumor treatment, influencing therapeutic responses and contributing to drug resistance^[1,9,10]^.

The advent of single-cell and spatial omics technologies has transformed the study of tumor complexity by enabling the exploration of intratumoral differences with unprecedented resolution^[7,11]^. These tools allow precise measurement of genomic, transcriptomic, epigenomic, and proteomic features at single-cell or spatial-spot resolution^[2,7,12-19]^. This capability enables high-resolution mapping of tumor cellular composition, the prediction of evolutionary pathways, and the characterization of complex intercellular interaction networks^[20-22]^. Consequently, these analyses further reveal the pronounced internal heterogeneity of tumors and their functional implications.

Despite the availability of diverse cytotoxic, immunotherapeutic, and targeted agents, drug resistance remains a major barrier to effective cancer treatment and significantly affects patient outcomes and quality of life^[1,11]^. Extensive research shows that tumor heterogeneity drives tumor evolution, shapes therapeutic responses, and ultimately contributes to sustained drug resistance^[23-25]^. Therefore, a comprehensive understanding of tumor heterogeneity is essential not only for elucidating tumor initiation, progression, and evolution, but also for uncovering drug resistance mechanisms, identifying therapeutic targets, and guiding the development of personalized treatment strategies.

In this review, we systematically outline emerging and established mechanisms by which tumor heterogeneity and evolution contribute to drug resistance across diverse therapeutic modalities. We further integrate current knowledge on the molecular mechanisms underlying targeted therapy failure. To address this complexity posed by tumor heterogeneity, we highlight how recent advances in single-cell and spatial multi-omics technologies provide the resolution required to map resistant subclones and their spatial niches. Building on these developments, we propose a dynamic, biology-driven clinical workflow. Our central premise is that static treatment models are inherently insufficient against evolving tumors. By continuously monitoring tumor evolution and integrating multi-omics analyses with personalized functional validation, we outline practical strategies to anticipate resistance and maintain therapeutic efficacy.

## MECHANISMS OF DRUG RESISTANCE DRIVEN BY TUMOR HETEROGENEITY

### Evolutionary dynamics of tumor drug resistance

**Figure 2:**
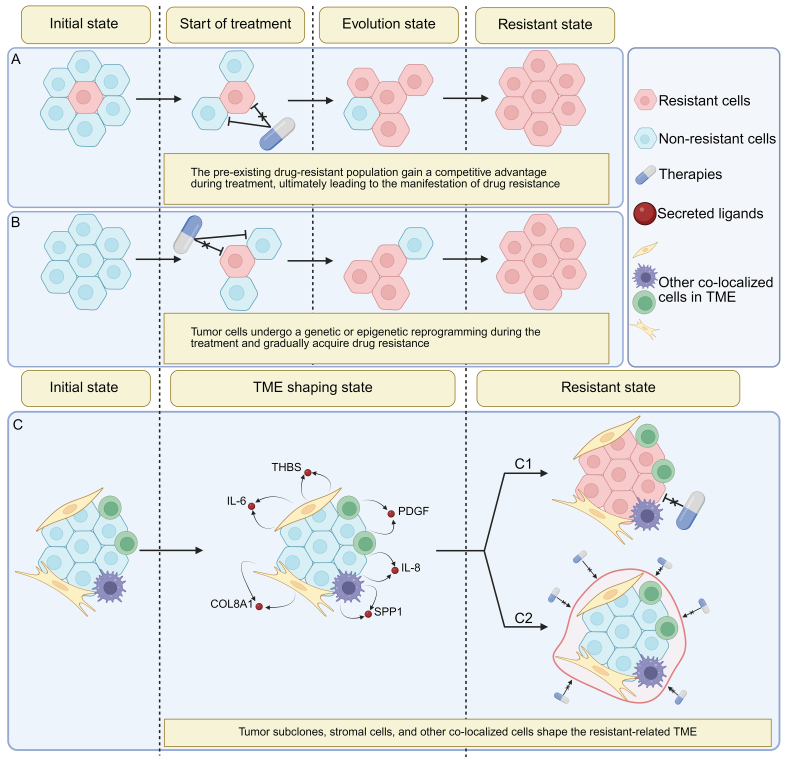
Multilevel evolutionary mechanisms of therapy resistance driven by tumor heterogeneity. (A) Pre-existing resistant clones. Prior to treatment, tumors harbor specific resistant clones that survive under drug pressure, gradually gain dominance, and ultimately manifest as tumor resistance; (B) Adaptive evolution. Under therapeutic pressure, tumors progressively alter their multi-omics features, developing and amplifying resistant clones, which ultimately lead to treatment failure; (C) Changes in the local TME. Tumor cells extensively communicate with surrounding cells within the specific TME in which the tumor is established, thereby shaping a resistance-associated microenvironment. (C1) The drug-resistant TME drives tumor cells to evolve drug-resistant phenotypes. (C2) The drug-resistant TME forms specialized barriers that impede drug efficacy. Created in BioRender. Da, E. (2026) https://BioRender.com/7257tar. TME: Tumor microenvironment; COL8A1c collagen VIII alpha1 chain; IL-6: interleukin-6; IL-8: interleukin-8; PDGF: platelet-derived growth factor; SPP1: secreted phosphoprotein 1; THBS: thrombospondin.

Tumor drug resistance is an evolutionary process driven by Darwinian selection, branched evolution, and microenvironmental constraints. Under the pronounced selection pressure of therapeutic interventions, resistant populations emerge through distinct evolutionary pathways that can be broadly categorized into selection of pre-existing resistant clones, adaptive evolution, and changes in the local TME. During selection of pre-existing resistant clones, a subset of tumor cells already possesses genetic or epigenetic features that confer a survival advantage^[26]^. Upon drug exposure, clones bearing resistance-associated alterations preferentially survive and expand, eventually becoming the dominant population and conferring drug-resistant phenotypes to the tumor^[26-29]^. In contrast, during adaptive evolution, multiple tumor clones initially exist in a relatively equivalent state. Under therapeutic pressure, most cells are eliminated, whereas a small fraction acquires genetic or epigenetic changes. These changes allow them to escape dependence on the drug target, ultimately giving rise to newly resistant tumor subpopulations^[26]^. Beyond intrinsic alterations within tumor cells, the TME can also promote resistance through tumor-stroma signaling, tumor–tumor communication, and diverse microenvironmental states co-established by tumor cells and stromal elements^[30-32]^ [Figure 2]. The selection of pre-existing clones, adaptive reprogramming, and microenvironmental remodeling collectively contribute to drug resistance and therapeutic failure across chemotherapy, targeted therapy, and immunotherapy.

#### Pre-existing resistant clones

Tumors may harbor pre-existing drug-resistant genotypes or epigenetic phenotypes that gain a competitive advantage during treatment, ultimately leading to the manifestation of drug resistance. For example, a study found that before gefitinib treatment in epidermal growth factor receptor (EGFR)-mutated lung cancer, a cell subpopulation with amplification of the oncogene mesenchymal-epithelial transition factor (MET) was already present in the tumor. This subpopulation developed resistance to gefitinib when activated by hepatocyte growth factor (HGF) through the phosphatidylinositol 3-kinase (PI3K)/protein kinase B (AKT)/GRB2-associated binding protein 1 (GAB1) signaling pathway after drug-related selection^[33]^. In an evolutionary model, events such as MET amplification can originate from a rare pre-existing subclone generated by neutral drift or early branched evolution. Upon EGFR-tyrosine kinase inhibitor (TKI) treatment, this specific subclone undergoes a pronounced clonal sweep driven by strict Darwinian selection, rapidly expanding to dominate the bulk of the tumor and manifesting as clinical resistance. Studies on leukemia have also indicated that pre-existing resistant subpopulations exist before treatment^[25]^. Prior to therapy, certain leukemia subclones exhibit chromatin accessibility–mediated epigenetic upregulation of T-cell lymphoma invasion and metastasis-inducing protein 1 (TIAM1) and zinc finger protein 257 (ZNF257) promoters, conferring enhanced resistance^[25]^. Similarly, in chronic lymphocytic leukemia (CLL), a latent ibrutinib-resistant subclone was identified before treatment. This subclone displayed MYC target gene upregulation, marked C-X-C motif chemokine receptor (CXCR)4 downregulation, and breakpoint cluster region (BCR) pathway activation, resembling lymph-node-like activated cells^[34]^. During therapy, this pre-existing subclone underwent selective expansion, ultimately leading to ibrutinib resistance at relapse^[34]^.

In triple-negative breast cancer (TNBC), reprogramming of histone H3K27me3 sites and DNA methylation–mediated chromatin alterations may drive resistance to 5-fluorouracil (5-FU) and capecitabine^[27]^. Throughout this process, intratumoral diversity progressively decreases, indicating pressure-driven selection among the initial subclonal populations^[27]^. Resistant subclones subsequently emerge as dominant components of the drug-resistant tumor^[27]^. Likewise, in muscle-invasive bladder cancer (MIBC), two distinct resistant subclones,S1 and S4,were already present before neoadjuvant immune checkpoint inhibitor (CPI) therapy^[35]^. S1 was enriched for fibroblast growth factor receptor 3 (FGFR3)/Kirsten rat sarcoma viral oncogene homolog (KRAS) mutations and peroxisome proliferator-activated receptor gamma (PPARG) amplification, whereas S4 showed upregulation of MYC, cell cycle, and DNA damage response (DDR)-related pathways^[35]^. These subclones constituted the initial resistance population and gained a competitive advantage during treatment, representing a major cause of pembrolizumab resistance^[35]^.

A study on sonic hedgehog (SHH)-type medulloblastoma revealed significant intratumoral spatial heterogeneity in pediatric medulloblastoma with chromothripsis^[36]^. These tumors displayed enhanced proliferative and stem-like features with reduced immune infiltration, and contained two major drug-resistant clones: Clone A, an “ancestral” near-diploid clone, and Clone B, which underwent additional chromothripsis, exhibiting polyploidy and complex chromosomal rearrangements^[36]^. These two clones coexisted across tumor regions both before and during treatment, thereby driving disease recurrence^[36]^.

#### Resistance acquired by reprogramming

Tumors may undergo genetic or epigenetic reprogramming during treatment and gradually acquire drug resistance. Before the acquisition of permanent genetic mutations, cancer cells often exploit non-genetic plasticity to enter a reversible, drug-tolerant persister (DTP) state^[28]^. This early epigenetic reprogramming serves as a vital survival reservoir, allowing persister cells to withstand initial therapeutic pressure until more permanent resistance mechanisms evolve^[37]^.

In the study on MIBC discussed earlier, in addition to the selection of pre-existing subclones (S1 and S4) the same cohort also exhibited adaptive evolution during pembrolizumab treatment. Specifically, therapeutic pressure induced the emergence of two novel subclones, S6 and S7, which were entirely absent prior to therapy^[35]^. S6 subclone activated the FGFR–mitogen-activated protein kinase (MAPK) pathway and upregulated transforming growth factor (TGF)-β1, SMAD2/3, zinc finger E-box binding homeobox 1 (ZEB1), Snail family transcriptional repressor 2 (SNAI2), and other transcription factors through histone deacetylation and methylation reprogramming, exhibiting epithelial–mesenchymal transition (EMT)-like characteristics. The S7 subclone, on the other hand, activated inflammatory signals such as interferon-gamma (IFN-γ), tumor necrosis factor-alpha (TNF-α), and interleukin (IL)-6, upregulated the MAPK/MAPK-ERK kinase (MEK)/protein kinase C (PKC) pathway, and demonstrated immune-tolerant properties^[35]^.

Measurable residual disease (MRD) after chemotherapy is a common cause of resistance and relapse in pediatric B-cell acute lymphoblastic leukemia (B-ALL)^[23]^. Zhang *et al.* employed paired single-cell B-cell receptor sequencing (scBCR-seq) and single-cell RNA sequencing (scRNA-seq) in pediatric B-ALL patients treated with first-line conventional chemotherapeutic agents (cytarabine, daunorubicin, and vincristine) and used these paired single-cell approaches to track temporal tumor heterogeneity^[23]^. The authors integrated data from three phases of disease progression in the same patient - diagnosis (Dx), day 19 of induction chemotherapy (D19; MRD), and relapse (Rel) - and identified distinct phenotypic differences across these phases. D19 samples exhibited widespread G0/G1 arrest, with differential enrichment analyses revealing significantly upregulated hypoxia-related pathways. In contrast, Rel samples showed a shift toward less differentiated or more primitive leukemic states, with increased proportions of earlier-stage components - such as pro-B or hematopoietic stem cell (HSC)/lympho-myeloid primed progenitor (LMPP)-like cells - and resolution of the G0/G1 arrest observed at D19^[23]^. These findings indicate that, under chemotherapeutic pressure, tumor cells collectively transitioned from their original Dx state to a stress-response and survival state at D19, and subsequently to a low-differentiation, re-proliferative state in Rel^[23]^. This temporal reprogramming ultimately led to chemotherapy failure.

Using scRNA-seq, Miao *et al.* reported that in squamous cell carcinoma (SCC) a population of TGF-β-responsive α6hi/cluster of differentiation (CD) 34^+^/CD44^+^ tumor-initiating stem cells (tSCs) selectively acquired high CD80 expression to preferentially survive adoptive cytotoxic T-cell transfer (ACT)-based immunotherapy and drive recurrence^[38]^. During this process, CD80 expressed by tumor cells bound directly to cytotoxic T-lymphocyte-associated protein 4 (CTLA4) on cytotoxic T lymphocytes (CTLs), and inhibited CD8^+^ T-cell proliferation and effector molecule production,such as granzyme B (GZMB), IFN-γ, and TNF-α, thereby promoting T-cell exhaustion and establishing adaptive immune tolerance^[38]^. Correspondingly, knocking out CD80 or blocking CTLA4 restored CTL activity, increased tSC apoptosis, and reduced recurrence^[38]^. Similarly, in multiple myeloma, resistance to bispecific antibodies targeting G-protein-coupled receptor family C group 5 member D (GPRC5D) could be gained through gene inactivation or through long-range epigenetic silencing of its promoter and enhancer regions^[39]^. Whole-genome duplication (WGD) also plays a significant role in the development of acquired drug resistance in tumors. In the TRAcking Cancer Evolution through therapy (Rx) (TRACERx) project, researchers found that in many cases of lung SCC and adenocarcinoma, cancer cells first undergo extensive loss of heterozygosity (LOH), followed by WGD^[40]^. This allows them to evade the control of tumor suppressor genes and also the lethal consequences of defects in survival-related genes, demonstrating the sophisticated strategies employed by tumor cells to gain drug resistance.

#### Changes in the local TME

Specific tumor subclones, stromal cells, and other cellular components can shape distinct TMEs, thereby generating local conditions that ultimately contribute to drug resistance. In the interaction between the TME and drug therapy, the TME frequently alters the entire tumor tissue’s response to drug treatment through mechanisms such as rebuilding the extracellular matrix (ECM) physical barrier, reshaping the immunosuppressive microenvironment, and modulating paracrine signaling.

In urothelial carcinoma treated with gemcitabine plus cisplatin, Kikuchi *et al.* observed that drug-stimulated tumor cells upregulated IL-8 secretion, which in turn acted on tumor endothelial cells to activate nuclear factor kappa-light-chain-enhancer of activated B cells (NF-κB)^[41]^. This activation led to increased ATP-binding cassette sub-family B member 1 (ABCB1) expression in tumor endothelial cells, conferring drug-efflux capacity that not only enabled tumor tissues to resist first-line chemotherapeutic agents such as gemcitabime and cisplatin, but also reduced the efficacy of second-line drugs such as paclitaxel^[41]^.

Under the selective pressure of immunotherapy, the tumor immune microenvironment undergoes extensive immunoediting. Tumor sites with low intrinsic resistance and poor immunogenicity are preserved due to their strong survival capacity, allowing them to establish a “cold” TME that ensures their long-term persistence. In metastatic melanoma, immune cells and cancer-associated fibroblasts (CAFs) jointly shaped a microenvironment resistant to anti-programmed cell death protein 1 (PD-1) and anti-CTLA-4 immunotherapy. In this microenvironment, CD8^+^ T cells decreased in number and accumulated at the tumor periphery, whereas B cells and plasma cells became enriched within the tumor and formed lymphoid follicle-like structures^[42]^. At the same time, CAFs upregulated chemokines and genes associated with ECM remodeling, thereby establishing both physical and immune barriers^[42]^. Similarly, intraductal papillary mucinous neoplasms (IPMN), the precursor lesions of pancreatic ductal adenocarcinoma (PDAC), also exhibited an immunosuppressive TME^[43]^. High-grade IPMN was found to secrete large amounts of abnormally O-glycosylated mucins that formed physical and immunosuppressive barriers. These impeded natural killer (NK)/T-cell recognition and infiltration and promoted immune escape, ultimately leading to resistance to the combination of gemcitabine and nab-paclitaxel^[43]^. Liu *et al.* also identified specific tumor immune barrier (TIB) structures at the tumor periphery during immune checkpoint inhibitor (ICI) treatment of hepatocellular carcinoma (HCC)^[44]^. These structures manifested as secreted phosphoprotein 1-positive (SPP1^+^) macrophage-CAF colocalization, in which SPP1^+^ macrophages highly expressed ligands such as TGFB1, SPP1, and interleukin-1beta (IL1B), while CAFs highly expressed their corresponding receptors. Subsequent activation of downstream collagen genes (*COL1A1*/*2*/*3A1*/*4A1*/*5A1*), matrix metalloproteinases (MMP) and their inhibitors, and chemokines [C-C motif chemokine ligand (CCL) 3/4/5, CXCR4] led to regulation of ECM organization, cell adhesion, fibrotic responses, and chemotactic signaling, and ultimately to resistance to CD8^+^ T-cell infiltration^[44]^.

The types and frequency of cell-cell interactions between stromal cells and tumor cells are also dynamically reshaped under therapeutic pressure, resulting in connections that are more conducive to tumor survival. Zhou *et al.* reported that in colorectal cancer samples from patients receiving oxaliplatin chemotherapy, thrombospondin-2 (THBS2) positive CAFs localized close to oxaliplatin-resistant malignant cells and engaged in collagen-mediated interactions^[45]^. THBS2^+^ CAFs specifically secreted COL8A1, which directly bound to integrin subunit beta 1 (ITGB1) on the surface of resistant malignant cells, activated the PI3K-AKT pathway, induced EMT, and ultimately led to drug resistance^[45]^. Additionally, by integrating The Cancer Genome Atlas Program (TCGA) bulk RNA-seq data, pan-cancer protein datasets, pan-cancer cell line expression data, pan-cancer scRNA-seq data, and public spatial transcriptomics datasets from ovarian, breast, pancreatic, and head and neck squamous cell carcinomas (HNSCC), THBS was found to be enriched in CAFs across multiple cancer types, suggesting that such tumor-stroma interactions might be associated with poor chemotherapy response across a broad spectrum of cancers^[45]^. Similarly, carboplatin-resistant ovarian clear cell carcinoma (OCCC) subclusters frequently colocalized with CAFs, forming a unique resistance niche in which tumor cell subclusters activated CAFs via platelet-derived growth factor (PDGF). These activated CAFs, in turn, induced tumor cells to upregulate hypoxia inducible factor (HIF)-1α/HIF-2α, thus promoting chemotherapy resistance^[46]^. In drug-resistant breast cancer gene (*BRCA*)*1/2*-mutated breast cancer treated with selective poly (ADP-ribose) polymerase inhibitors (PARPi) like olaparib and AZD5305, a specific tumor-associated macrophage (TAM) subset, TAM_C3, was enriched within the TME. These macrophages exhibited enhanced 40S ribosomal protein S19 (Rps19)–complement component 5a 1eceptor 1 (C5aR1) signaling with tumor cells, thereby suppressing effector molecules such as GZMB and perforin 1 (PRF1) in CD8^+^ T cells, inducing immune suppression, and ultimately promoting resistance to PARPi therapy^[47]^. Similarly, during osimertinib treatment for non-small cell lung cancer (NSCLC), M2-TAM exosomes carrying lncRNA MSTRG.292666.16 were upregulated and delivered to tumor cells, thus activating the MSTRG.292666.16–miR-6836-5p–C-jun-amino-terminal kinase-interacting protein 3 (MAPK8IP3) axis and consequently activating MAPK signaling. The resulting enhanced tumor cell survival and growth in the presence of osimertinib led to the development of acquired resistance^[48]^.

The TME functions as a dynamic system that co-evolves with tumor clones via bidirectional signaling and metabolic coupling, rather than acting as a static barrier. In study using osimertinib to treat NSCLC, Zhang *et al.* identified a collagen triple helix repeat containing 1 (CTHRC1) positive CAF subpopulation that promoted TGF-β1 binding to TGF beta receptor 2 (TGFBR2) by secreting CTHRC1, thereby enhancing cancer cell glycolysis through the TGF-β/SMAD3/hexokinase 2 (HK2) axis and leading to EGFR-TKI resistance^[49]^. Conversely, tumor cells increased lactate concentrations in the TME through glycolysis, which in turn upregulated CTHRC1 expression in CAFs via p300-mediated H3K18la, thus completing a positive feedback loop^[49]^.

Treatment-induced permanent cell cycle arrest in cancer cells is known as therapy-induced senescence (TIS) and is often regarded as a highly successful outcome of cancer therapy because it irreversibly halts the proliferation of malignant cells^[50]^. However, these senescent cells remain metabolically hyperactive. They secrete a complex proteome comprising pro-inflammatory cytokines (such as IL-6, IL-1α/β), chemokines [such as C-X-C motif chemokine ligand (CXCL) 8 and 11], MMPs, and small extracellular vesicles (sEVs), collectively known as the senescence-associated secretory phenotype (SASP)^[50,51]^. The SASP shows dynamic changes over the course of cancer treatment. Under prolonged therapeutic pressure, the chronic SASP signals shift from an initial tumor-suppressive role to a highly pro-inflammatory and matrix-degrading effect, becoming one of the primary contributors to drug resistance and tumor recurrence^[51]^. For example, Raynard *et al.* found in breast and prostate cancer models that NF-κB-dependent SASP factors secreted by senescent cells can directly induce neuroendocrine transdifferentiation in adjacent epithelial cancer cells, endowing them with strong invasiveness and intrinsic resistance to treatment^[52]^. These findings illustrate the dynamic changes in TME that occur during therapy, gradually leading to the development of a treatment-tolerant phenotype.

### Targeted therapy resistance in heterogeneous tumors

**Figure 3:**
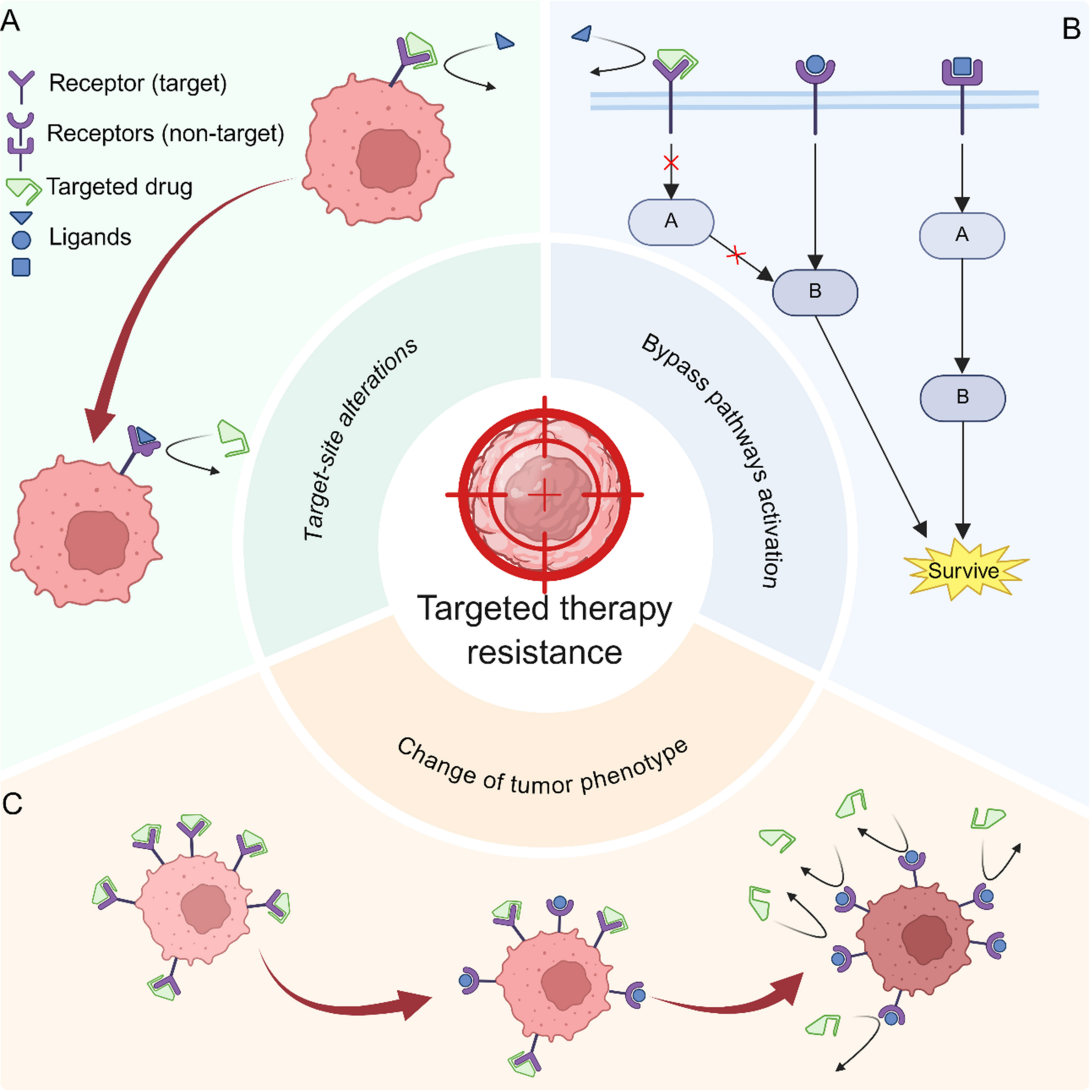
Three primary mechanisms of resistance to tumor-targeted therapy. (A) Modifications or alterations to the target itself can impair the specificity of targeted drugs; (B) Following inhibition of the tumor target, downstream signaling pathways may be activated by compensatory alternative routes. Tumors may also rely on the compensatory activation of other survival-related pathways to offset the effects of blocked target-associated signaling; (C) Tumors may undergo a series of phenotypic changes and acquire additional traits that enable them to become completely independent of the target, thereby rendering the original targeted therapy ineffective. Created in BioRender. Da, E. (2026) https://BioRender.com/ryuh0ks.

Targeted cancer therapy typically leverages molecular abnormalities in tumors, such as driver gene mutations, amplified receptor tyrosine kinases, or activated key signaling pathways, by employing small-molecule inhibitors or monoclonal antibodies to selectively interfere with these abnormal pathways^[53,54]^. Although this approach achieves a higher therapeutic index and enables personalized treatment strategies, tumor heterogeneity poses significant challenges to treatment efficacy^[55]^ [Figure 3]. While evolutionary dynamics provide a general framework for resistance, targeted therapies involve additional complexity due to their specific molecular dependencies. Beyond these universal evolutionary principles, alterations at the level of target engagement and signaling are the ultimate drivers of acquired resistance to targeted therapies.

#### Target site alterations

Genetic mutations or epigenetic modifications occurring at the target site itself may alter the target’s expression or conformation, thereby preventing the drug from effectively binding to its intended target.A classic example of this type of targeted therapy resistance mechanism is the EGFR-TKI-based targeted therapy for NSCLC. A large number of off-target mutations have been identified in EGFR to date, with considerable interpatient variability, including C797S^[56-60]^, G796S/R/D^[61-63]^, L792 and L718 mutations^[61,64]^, G724S^[65-67]^, and EGFR amplification^[68,69]^. Heterogeneity adds an extra layer of complexity: different subclones within the same patient may harbor diverse resistance mutations simultaneously. For example, during crizotinib therapy, patients exhibited different resistance mechanisms, and even a single patient could harbor multiple resistance-associated mutations^[70]^.

BRAF inhibitors such as vemurafenib or dabrafenib are commonly used to treat melanoma harboring BRAF V600E/K mutations. However, BRAF amplification and abnormal alternative splicing can confer resistance to the cytotoxic effects of BRAF inhibitors by increasing BRAF kinase activity levels or generating truncated BRAF variants that are more prone to dimerization, with the types of resistance varying among different patients^[71-73]^. In targeted therapies for chronic myeloid leukemia involving BCR–Abelson murine leukemia viral oncogene homolog (ABL) inhibition, different BCR–ABL mutant clones, such as Y253H, E255K/V, F317L, and T315I, may coexist, or even form compound mutations, and be selected under drug pressure, thereby leading to resistance^[74,75]^. The resistance patterns also vary with specific mutations. For example, Y253H and E255K/V were resistant to imatinib but remained sensitive to dasatinib; F317L was resistant to both imatinib and dasatinib but was sensitive to nilotinib; and T315I conferred resistance to all three inhibitors^[74,75]^.

#### Activation of bypass pathways

By inducing genetic or epigenetic alterations in bypass pathways or downstream targets, bypass activation or downstream reactivation can be established in tumor subclones, leading to targeted drug failure. Using targeted therapy for NSCLC as an example, bypass activation, including abnormal amplification of MET and the human EGFR (HER2), has long been recognized as one of the mechanisms underlying resistance to EGFR-TKIs^[76,77]^. When EGFR was inhibited, its downstream signaling pathways, particularly the PI3K/AKT and MAPK pathways, could still be activated via MET or HER2, thereby bypassing EGFR dependency and leading to treatment failure with EGFR-TKIs^[78]^. Similarly, when EGFR-mutated NSCLC was treated with the third-generation EGFR-TKI osimertinib, some cells did not undergo cell death but instead entered a DTP state^[79]^. These cells exhibited unique characteristics, including slowed proliferation, reduced dependence on EGFR signaling, and enhanced antiapoptotic capacity^[79]^. In DTP cells Izumi *et al.* discovered that B-cell lymphoma 2-like protein 1 (*BCL2L1*) was significantly upregulated, whereas pro-apoptotic genes such as induced myeloid leukemia cell differentiation protein (*MCL1*), BCL2-associated X protein (*BAX*), and BCL2 homologous antagonist/killer 1 (*BAK1*) were downregulated. This enabled resistance to EGFR-TKI–induced apoptosis and prolonged survival, which in turn provided a time window for the accumulation of new mutations in drug-resistant clones^[79]^. Conversely, knockout or pharmacological inhibition of BCL2L1 restored NSCLC sensitivity to osimertinib, suggesting its potential as a therapeutic target^[79]^.

During tamoxifen treatment, breast cancer acquired a novel epigenetic landscape characterized by loss of H3K27me3 at polycomb-group (PcG) protein-targeted genes and genes associated with basal-like mammary epithelial signatures driven by the selective evolution of pre-existing drug-resistant subclones. These alterations led to the upregulation of genes such as *EGFR*, insulin-like growth factor binding protein 3 (*IGFBP3*), and activated leukocyte cell adhesion molecule (*ALCAM*), established novel survival and proliferation pathways, and ultimately drove the emergence of tamoxifen resistance^[80]^.

Bevacizumab is an anti-vascular endothelial growth factor (VEGF) pathway antibody. During bevacizumab therapy for glioblastoma (GBM), reactive activation of MET-related signaling pathways occurred, thereby leading to targeted treatment resistance^[81]^. Using paired tumor specimens from GBM patients before and after bevacizumab treatment, together with a mouse intracranial GBM model, Lu *et al*. demonstrated that prior to treatment, VEGF recruited the protein tyrosine phosphatase PTP1B by binding to the MET/VEGF receptor 2 (VEGFR2) heterodimer complex, thereby inhibiting HGF-induced tyrosine phosphorylation of MET and cell migration^[81]^. When VEGF was blocked, this inhibitory mechanism was relieved, thereby activating MET-related pathways and triggering EMT, which resulted in increased invasiveness, distant metastasis, and targeted therapy resistance^[81]^. Similarly, when gefitinib and erlotinib were used as EGFR-TKIs to treat GBM, targeted resistance accompanied by MET activation also occurred^[82]^. Jun *et al.* found that activated MET sustained downstream survival signaling, such as AKT, thereby shifting the response to EGFR-TKIs from cytotoxicity to cell quiescence (G1 arrest) and conferring tolerance^[82]^. In contrast, combined inhibition of EGFR and MET restored apoptosis and reduced survival, overcoming this tolerant phenotype^[82]^.

#### Change of tumor phenotype

Tumor tissues may undergo various phenotypic alterations, such as EMT or metabolic reprogramming, during subclone variation and selection, rendering targeted drugs ineffective. As observed in the aforementioned study on MIBC, drug resistance can manifest through phenotypic changes. Specifically, the emergent S6 subclone displayed EMT features in tumor tissues and showed associated drug resistance^[35]^. Smoothened inhibitors (SMOi) target the Hedgehog pathway and are commonly used as first-line treatments for basal cell carcinoma (BCC) associated with Gorlin syndrome (nevus BCC syndrome)^[83]^. Sporadic BCC exhibits a high rate of resistance, whereas tumors arising in Gorlin syndrome patients with germline patched-1 (PTCH1) mutations show uniform suppression with inhibitor therapy^[83]^. Jussila *et al.* combined 10× Genomics scRNA-seq with 10× Genomics Visium spatial transcriptomics to investigate why a small subset of Gorlin syndrome patients treated with long-term inhibitors developed drug-resistant tumor clones and progressed rapidly^[83]^. They found that tumors in resistant Gorlin syndrome patients underwent basal-to-squamous transdifferentiation (BST), building SCC-like regions that exhibited a Hedgehog-independent phenotype^[83]^. During this process, GLI family zinc finger 1 (GLI1) signaling was significantly downregulated, whereas the phosphatidylethanolamine synthesis pathway was activated. Mutations in the tumor regions undergoing BST were found enriched in phosphate cytidylyltransferase 2 (PCYT2) and ethanolamine kinase 1 (ETNK1), ultimately leading to increased expression of squamous differentiation marker genes, such as cytokeratin 5/6 (*KRT5/6*), tumor protein p63 (*TP63*), and lymphocyte antigen 6 family member D (*LY6D*), in tumor cells. This resulted in a SCC phenotype, loss of dependence on SMO signaling, and subsequent targeted therapy resistance^[83]^. Similarly, in BCC treated with SMOi, Li *et al.* used scRNA-seq, single-cell assay for transposase-accessible chromatin sequencing (scATAC-seq), and cleavage under targets and release using nuclease (CUT&RUN) to identify and define an NF-κB-driven tumor epithelial state known as the basal-to-inflammatory transition (BIT)^[84]^. During this process, triggering receptor expressed on myeloid cells 1 (TREM1) positive myeloid cells within a specialized intratumoral niche secreted IL-1 and oncostatin M (OSM), activating the NF-κB/signal transducer and activator of transcription 3 (STAT3) pathway in the tumor epithelium, directly upregulating BIT markers such as chitinase-3-like protein 1 (CHI3L1) and vascular cell adhesion molecule-1 (VCAM1) while downregulating Hedgehog/GLI1 signaling, thereby reducing sensitivity to SMOi^[84]^. Notably, Li *et al.* detected both BIT and BST sites coexisting in a non-overlapping manner: BIT resided in the inflammation-enriched, myeloid-dominated superficial-epithelial niche, whereas BST was more tumor-core-oriented and matrix-isolated^[84]^. This further highlights the complexity of tumor-intrinsic heterogeneity and demonstrated how local heterogeneity within the tumor–immune environment shapes drug responses.

Some EGFR-mutated lung adenocarcinomas (LUAD) undergo transformation into small cell lung cancers (SCLC) after EGFR-TKI treatment, resulting in drug resistance^[85]^. Using 10× Visium-FFPE, Li *et al.* revealed that neurodifferentiation-related pathways were upregulated during this process, whereas NSCLC-specific, apoptosis, cell-adhesion, and immune-related pathways were downregulated^[85]^. This was accompanied by reduced histone deacetylase 1 (HDAC10) expression and sustained activation of fibroblast growth factor (FGF) signaling pathways^[85]^. Consequently, the epidermal growth factor (EGF)–EGFR axis and its downstream signaling were significantly attenuated following transformation, whereas the FGF–FGFR axis was markedly upregulated and maintained sustained activity consistent with pathway dependency^[85]^. This allowed tumor cells to escape EGFR dependency and resulted in resistance to EGFR-TKIs.

## SINGLE-CELL AND SPATIAL PROFILING OF RESISTANCE PATHWAYS

**Figure 4:**
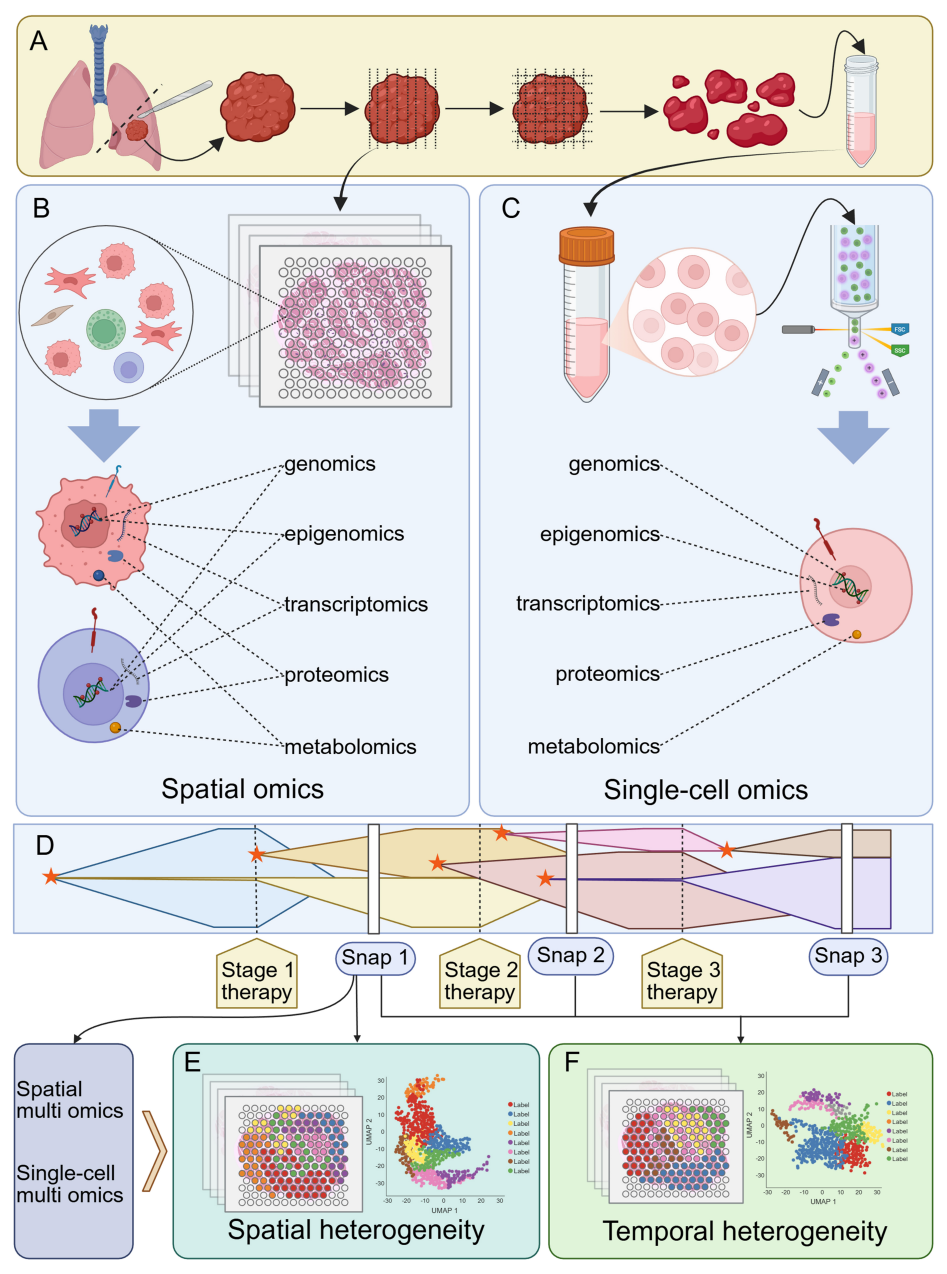
Investigating tumor resistance mechanisms associated with heterogeneity using single-cell and spatial multi-omics technologies. (A) Tumor tissue processing workflow. Tumor tissue obtained via surgical resection or other methods can be processed into slices for spatial multi-omics analysis, whereas tissue lysates yielding single-cell suspensions can be used for subsequent single-cell multi-omics analysis; (B) Spatial multi-omics analysis. Tissue slices are segmented into multiple spatial spots, with all cells within each spot undergoing comprehensive multi-omics profiling; (C) Single-cell multi-omics analysis. Single-cell suspensions undergo multi-step sorting and processing, ultimately enabling cell-level multi-omics profiling; (D) Diagram illustrating tumor heterogeneity during development. Tumor evolution involves multiple subclonal formation processes, with different subclones coexisting and undergoing selection and evolution under drug pressure or other survival stresses. Throughout this process, tumor tissue can be sampled at multiple time points (snaps) and analyzed using spatial or single-cell multi-omics approaches. A single snap data point addresses spatial tumor heterogeneity (E), whereas combined analysis of multiple data points addresses temporal tumor heterogeneity (F). The asterisks indicate subclonal formation. Created in BioRender. Da, E. (2026) https://BioRender.com/5zem2e5.

Despite their contributions to understanding tumorigenesis, progression, and treatment response, bulk omics technologies lack the resolution necessary to capture cellular and regional heterogeneity, including rare and spatially organized subclonal mechanisms that underlie drug resistance^[86,87]^. Closing this gap requires advanced high-resolution analytical tools capable of resolving tumor heterogeneity. The emergence of single-cell multi-omics and spatial multi-omics technologies has provided researchers with powerful tools to dissect intratumoral heterogeneity at the single-cell or spatial-spot level^[88-91]^ [Table 1]. These technologies have enabled more precise investigations of tumor resistance mechanisms and informed corresponding therapeutic strategies^[88-91]^ [Figure 4].

**Table 1:** Summary of single-cell and spatial multi-omics technologies for profiling tumor heterogeneity and resistance

**Category**	**Technology**	**Characteristics**	**Ref.**
Single-cell transcriptomics and genomics	10× Genomics scRNA-seq	A microfluidic droplet-based, high-throughput, multi-sample parallel scRNA-seq technology	[15]
Single-cell epigenomics	ATAC-seq/snATAC-seq/sc-ATAC-seq	A technology that utilizes the hyperactive bacterial transposase Tn5 to analyze nucleosome-free or nucleosome-depleted regions within the genome and assesses chromatin accessibility based on DNA regulatory elements	[16,92]
	scRRBS-seq	A CpG islands methylation status technology at the single-cell level	[93-96]
	scCGI-seq	A methylation-detection method that utilizes restriction enzymes	[97]
	scCHIP-seq	A single-cell chromatin immunoprecipitation sequencing technology	[80,98]
	scCUT&RUN/scCUT&Tag	*In situ* cleavage or tagging techniques based on micrococcal nuclease or Tn5-modified antibodies recognizing specific histone modification sites	[99,100]
	CyTOF, uCoTarget, and MAbID	Technologies that enable the simultaneous detection of multiple histone modification patterns. Not widely adopted yet	[101-103]
Single-cell proteomics	CITE-seq	A technology combining highly multiplexed protein-labeled detection with unbiased transcriptomic analysis of thousands of single cells	[104]
Single-cell metabolomics	SCMS	A technique for mass spectrometry analysis at single-cell resolution that is not limited by antigen-specific reagents and can profile thousands of proteins within a single cell	[105,106]
	sSRTC-scM	A single-cell mass spectrometry technique based on ILCEI-MS used to analyze metabolic heterogeneity and lipid metabolism alterations in resistant cells	[107]
Spatial transcriptomics and genomics	10× Visium Spatial Gene Expression	A chip-based spatial transcriptomics platform	[108,109]
	GeoMx DSP whole-transcriptome analysis	A technique that uses targeted FISH detection rather than direct sequencing of transcripts for spatial sequencing	[110,108]
	CosMx SMI single-cell spatial transcriptomics	A hybrid-based, single-molecule barcoded detection method for enzyme-free, nucleic acid amplification-free spatial sequencing	[111,112]
Spatial epigenomics	Spatial ATAC-seq	A technology that utilizes hyperactive bacterial transposase Tn5 *in situ* on tissue sections to generate spatial chromatin accessibility maps	[113-116]
	Spatial CUT&RUN/Spatial CUT&Tag	Spatial labeling technology based on micrococcal nuclease or Tn5-modified antibodies	[117]
Spatial proteomics	Nanostring GeoMx DSP	A technique for regionally assessing protein expression levels using specific probes	[110]
	Spatial CITE-seq	A proteomics technique that utilizes antibodies conjugated to oligonucleotides containing a polymerase chain reaction handle to recognize cellular proteins. It can simultaneously obtain mRNA transcriptomics and proteomics data at the same spot	[104,118]
	PhenoCycler/CODEX	A technique for quantifying protein expression using multiplex fluorescence microscopy	[119]
	LCM-MS-based spatial proteomics	A high-precision spatial proteomics method combining LCM and mass spectrometry	[120,121]
Spatial metabolomics	MALDI-FT-ICR IMS/MS	A technique that uses matrix-assisted laser desorption/ionization and Fourier transform ion cyclotron resonance mass spectrometry to perform laser scanning in a tissue section and obtain spatial metabolite abundance data	[122-124]

scRNA-seq: Single-cell RNA sequencing; ATAC-seq: assay for transposase-accessible chromatin sequencing; snATAC-seq: single-nucleus ATAC-seq; scATAC-seq: single-cell ATAC-seq; scRRBS-seq: single-cell reduced representation bisulfite sequencing; scCGI-seq: single cell CpG island sequencing; scCHIP-seq: single-cell chromatin immunoprecipitation sequencing; scCUT&RUN: single-cell CUT&RUN; scCUT&Tag: single cell CUT&Tag; CyTOF: cytometry by time-of-flight; uCoTarget: ultrahigh-throughput combined tagmenting enrichment for multiple epigenetic proteins; MabID: multiplexing antibodies by barcode identification; CITE-seq: cellular indexing of transcriptomes and epitopes by sequencing; SCMS: single-cell mass spectrometry; sSRTC-scM: surface-activated slow rate tandem charge single-cell MS; ILCEI-MS: intact living-cell electrolaunching ionization MS; DSP: digital spatial profiling; FISH: fluorescence *in situ* hybridization; SMI: spatial molecular imager; CUT&RUN: cleavage under targets and release using nuclease; CUT&Tag: cleavage under targets and tagmentation; CODEX: co-detection by indexing; LCM: laser capture microdissection; MS: mass spectrometry; MALDI-FT-ICR: matrix-assisted laser desorption/ionization Fourier transform ion cyclotron resonance; IMS: imaging mass spectrometry.

### Single-cell profiling of resistance pathways

Single-cell multi-omics technologies enable the interrogation of molecular features at cellular resolution, including genomics^[14,125]^, transcriptomics^[15,126]^, epigenomics^[16,93,94]^, proteomics^[17,127]^, and metabolomics^[13,128]^. Despite their immense potential, these technologies have inherent limitations, particularly in capturing rare resistant subclones. For instance, the high dropout rates and limited transcript capture efficiency of droplet-based scRNA-seq can obscure the detection of micro-subclones that initiate early resistance^[129,130]^. Consequently, the development of technologies with improved resolution, increased throughput, and enhanced detection efficiency is necessary.

#### Single-cell transcriptomics and genomics

Single-cell transcriptomics and genomics enable high-throughput mapping of gene expression and genetic variations, allowing precise identification of resistant subclones and their evolutionary pathways under therapeutic pressure. One of the most widely adopted single-cell transcriptomic approaches is 10× Genomics scRNA-seq^[15,131]^. For example, Jerby-Arnon *et al.* applied this technology, combined with *in situ* protein imaging, to melanoma treated with ICI. The authors identified an immune-resistant tumor-cell state characterized by high expression of cell cycle and transcription factor genes [cyclin-dependent kinase (CDK) 4/6–E2F–MYC axis], low expression of IFN-γ-response and major histocompatibility complex class I (MHC-I) pathways, a blunted response to IFN-γ stimulation, impaired antigen presentation, and absent chemotactic signaling, resulting in an immune-rejecting phenotype that ultimately led to ICI treatment failure^[132]^.

In docetaxel-resistant prostate cancer (CRPC), Schnepp *et al.* analyzed the parental and docetaxel-resistant DU145 and PC3 cell lines using scRNA-seq^[133]^. They identified coexisting sensitive and resistant regulatory states within the same cell line, accompanied by large-scale rearrangements of transcription factor-target gene connectivity in resistant cells, which were characterized by an increased G1-phase fraction and a decreased S/G2M-phase fraction^[133]^. Gene Ontology clusters related to cytoskeleton/mitosis, signaling, and metabolism were driven by distinct transcription factor combinations within the resistance networks^[133]^. Based on these findings, the researchers successfully overcame single-agent resistance by combining trichostatin A with docetaxel^[133]^. Similarly, Taavitsainen *et al.* combined scRNA-seq and scATAC-seq in enzalutamide (ENZ)-resistant CRPC and revealed extensive chromatin remodeling at open chromatin sites in post-treatment tumors, which initiated distinct transcriptional programs^[134]^. Among these cells, an ENZ-induced cluster closely associated with resistance displayed activation of the mechanistic target of rapamycin complex 1 (mTORC1) pathway, enhanced expression of MYC target genes, and re-expression of cell cycle drivers, thus leading to a therapy escape state^[134]^. As ENZ exposure extended to weeks or months, some clusters in the single-cell transcriptome began expressing neuroendocrine-like genes such as enhancer of zeste homolog 2 (*EZH2*), aurora kinase A (*AURKA*), paternally expressed 10 (*PEG10*), and SRY-box transcription factor 2 (*SOX2*), transitioning from androgen-dependent glandular-like cells to neuroendocrine-like lineages^[134]^. Under ENZ stimulation, CRPC progressively evolved from an initial short-term, induced-resistance phenotype to a long-term, consolidated-resistance phenotype, significantly compromising therapeutic efficacy^[134]^.

#### Single-cell epigenomics

Complementary to genetic profiling, single-cell epigenomics enables analysis of regulatory mechanisms associated with phenotypic plasticity and adaptive drug tolerance, beyond stable genetic alterations. Using scATAC-seq-based single-cell resolution determinations of chromatin accessibility, Satpathy *et al.* identified potential epigenomic drivers of T-cell exhaustion, a major cause of anti-programmed death-ligand 1 (PD-L1) immunotherapy failure^[135]^. Similarly, Derrien *et al.* performed single nucleus ATAC-seq (snATAC-seq) and RNA sequencing (RNA-seq) and found that multiple myeloma could gain resistance to bispecific antibodies targeting GPRC5D through genetic or epigenetic silencing of the corresponding gene^[39]^. Moreover, snATAC-seq also revealed that activation of AP-1 binding site–related motifs and enhancers, together with an increased proportion of mesenchymal cells, were primary drivers of drug resistance in recurrent GBM^[136]^.

The formation of 5-methylcytosine and its derivatives, hydroxymethylcytosine and formylcytosine, constitutes a major source of cancer epigenetic heterogeneity. Accumulation of 5-methylcytosine in CpG islands - promoter regions with high CpG content - inhibits gene expression. Similarly, accumulation within gene bodies or enhancers modulates cis-regulatory activity, leading to aberrant gene expression in cancer, with suppression of tumor suppressors, and activation of oncogenes^[137]^. Several techniques are already available that use single-cell reduced-representation bisulfite sequencing (scRRBS-seq) to detect the methylation status of multiple CpG islands at single-cell level^[93-96]^. Additionally, single cell CpG island sequencing (scCGI-seq), a methylation-detection method that utilizes restriction enzymes and is bisulfite-independent, has been developed and implemented^[97]^.

Beside DNA methylation and chromatin-accessibility regulation, histone modifications represent another major pathway of cellular epigenetic regulation^[27]^. A broad diversity of histone modifications exists in tumor cells, making single-cell approaches uniquely advantageous^[138]^. Single-cell chromatin immunoprecipitation sequencing (scCHIP-seq) provides valuable insights into single-cell level heterogeneity of histone modifications in tumor cells^[98]^. For example, Grosselin *et al.* utilized scSHIP-seq to reveal that breast cancer acquired resistance to capecitabine and tamoxifen during chemotherapy through selective evolution of pre-existing drug-resistant subclones and modifications at H3K27me3 sites^[80]^.

Additionally, at the single-cell level, *in situ* cleavage (CUT&RUN) or tagging (cleavage under targets and tagmentation, CUT&Tag) techniques enable single-cell histone modification analysis with improved signal-to-noise ratios and higher throughput^[99,100,139]^. Using CUT&Tag technology, specific cell populations can be detected based on chromatin states defined by histone modifications on adjacent nucleosomes^[138]^. Using this technology in gliomas, Wu *et al.* identified a Verhaak_GBM_proneural gene set that was specifically suppressed by PcG-mediated H3K27me3 in drug-resistant tumor subclusters. These subclusters were selectively enriched during treatment, driving tumor evolution toward a more drug-resistant mesenchymal phenotype^[139]^.

New technologies such as cytometry by time-of-flight (CyTOF), ultrahigh-throughput combined tagmenting enrichment for multiple epigenetic proteins (uCoTarget), and multiplexing antibodies by barcode identification (MAbID) enable the simultaneous detection of multiple histone modification patterns within cells with higher efficiency^[101-103]^. These approaches offer effective solutions for refined measurement and classification of tumor heterogeneity, as well as for investigating mechanisms underlying drug resistance.

#### Single-cell proteomics

Linking transcriptomic profiles to functional phenotypes requires single-cell proteomics, which enables detection of surface markers, signaling proteins, and post-transcriptional modifications involved in resistance mechanisms. In GBM, Pombo Antunes *et al.* combined cellular indexing of transcriptomes and epitopes by sequencing (CITE-seq) with scRNA-seq to systematically map spatiotemporal heterogeneity. They found that, following standard treatment with surgery plus radiotherapy and temozolomide (TMZ), the predominant population of microglia-derived TAMs (Mg-TAMs) was replaced by monocyte-derived TAMs (Mo-TAMs). This shift not only conferred TMZ resistance, but also established an innate tolerance foundation for resistance to PD-1/PD-L1 ICI^[140]^. Similarly, the combined use of CITE-seq and scRNA-seq in metastatic melanoma revealed that spatiotemporal heterogeneity of tumor and stromal cells jointly promoted resistance during combined anti-PD-1 and anti-CTLA-4 immunotherapy. More specifically, microphthalmia-associated transcription factor (MITF) positive, secreted protein acidic and rich in cysteine-like 1 (SPARCL1) positive and centromere protein F (CENPF) positive tumor subclones emerged and their proportion increased, reducing antigen-processing and antigen-presentation pathways while exhibiting EMT features^[42]^. Tian *et al.* utilized CITE-seq and revealed that pediatric GBM underwent selective amplification and immune escape under therapeutic pressure due to heterogeneous expression of tumor-associated antigens during chimeric antigen receptor T-cell (CAR-T) single-targeted therapy^[141]^. This led to insufficient CAR-T persistence and increased exhaustion, ultimately resulting in treatment failure^[141]^, Cadot *et al.* applied CITE-seq to CLL treated with the Bruton’s tyrosine kinase (BTK) inhibitor ibrutinib to identify a pre-treatment-existing, progression-enriched subclone characterized by BCR activation, MYC target gene upregulation, and CXCR4 downregulation^[34]^. The selective expansion of this subclone, coupled with BTK mutation accumulation and signaling pathway reprogramming, ultimately led to ibrutinib resistance^[34]^.

#### Single-cell metabolomics

Single-cell metabolomic approaches rely primarily on mass spectrometry (MS) and facilitate characterization of tumor cell biochemical phenotypes, supporting the investigation of drug resistance mechanisms associated with metabolic reprogramming. Irinotecan (IRI) is widely used in chemotherapy regimens for metastatic colorectal cancer (mCRC), but its efficacy is limited by acquired resistance^[106]^. Chen *et al.* analyzed the single-cell metabolomics of IRI-resistant HCT-116 cells using single-probe single-cell MS (SCMS)^[106]^. They found that the IRI-resistant subpopulation displayed cancer stem cell-like characteristics and significant metabolic reprogramming, including markedly upregulated sphingomyelins, downregulated tricarboxylic acid-cycle intermediates, and substantially elevated lactic acid^[106,142]^. Notably, lipid composition showed a marked increase in *de novo* fatty acid synthesis, a process directly correlated with fatty acid synthase (FASN) activity^[106]^. Similarly, combined metformin and IRI treatment significantly reduced FASN activity, leading to substantial downregulation of lipids such as phosphatidylcholine (PC), phosphatidylserine (PS), phosphatidylinositol (PI) and triglycerides (TG), and multiple fatty acids (e.g., palmitic, oleic and stearic acids), thereby restoring anti-CRC activity^[106]^. Sun *et al.* also applied SCMS to IRI-resistant CRC samples and identified lipid-metabolism reprogramming^[143]^. In IRI-resistant cells, multiple unsaturated phosphatidylcholines [PC(33:4), PC(34:4), PC(38:5), PC(34:3), PC(36:3), PC(34:2), PC(34:1), PC(36:6), *etc.*] were significantly elevated^[143]^. The total amounts of the major fatty acids C16:0, C16:1, C18:0, and C18:1 increased, and the ratios of monounsaturated to saturated fatty acids, including C16:1/C16:0 and C18:1/C18:0, were markedly elevated^[143]^. Further studies revealed that these changes were associated with significant upregulation of stearoyl-CoA desaturase-1 (SCD1) mRNA and protein in drug-resistant cells, resulting in elevated synthesis of unsaturated fatty acids, altered membrane fluidity and topology, reduced drug-lipid interactions, and impaired drug entry or membrane-associated activity^[143]^.

Similarly, Zhu *et al.* employed surface-activated slow rate tandem charge single-cell MS (sSRTC-scM), a single-cell MS technique based on intact living-cell electrolaunching ionization MS (ILCEI-MS), to analyze the mechanisms underlying secondary resistance to the first-generation EGFR-TKI gefitinib in EGFR-mutant NSCLC^[107]^. They found increased metabolic heterogeneity in PC9 cells during treatment, with gefitinib-resistant cells (PC9GR) showing lipid metabolism alterations centered on glycerophospholipid reprogramming, with significant increases in PC, PE, and sphingomyelin (SM)^[107]^. These changes reflected alterations in membrane lipid composition, signaling lipids, and lipid synthase activity, which were closely correlated with the resistant phenotype and increased with the degree of resistance^[107]^.

### Spatial profiling of resistance pathways

Spatial multi-omics technologies enable the simultaneous measurement of genomic^[2,144]^, transcriptomic^[18,118,145-153]^, epigenetic^[7,113,114,117,154-157]^, proteomic^[19,118,158-167]^, and metabolomic^[12,168]^ features across distinct spatial spots within a single tissue section at varying levels of precision, and were recognized as a key technology by *Nature* in 2022^[169]^. However, many current spatial transcriptomics platforms lack true single-cell resolution, resulting in transcriptomic data being pooled within spatial clusters and potentially obscuring rare, critical drug-resistant niches^[170,171]^. Meanwhile, the widespread adoption of single-cell resolution technologies is limited by cost. To accurately address these technical challenges, it is necessary to optimize sequencing depth and develop more affordable higher-resolution spatial technologies.

#### Spatial transcriptomics and genomics

Obtaining genotypes and gene expression patterns across distinct regions within an organ is crucial for identifying functional structures and their specialized roles within tumors. Unlike single-cell sequencing alone, integrating single-cell technologies with spatial omics enables more comprehensive analysis of cellular localization within tissues and the regulatory networks that shape cell–environment relationships^[91]^. For these reasons, spatial transcriptomics was selected by *Nature Methods* as “Method of the Year 2020”^[172]^.

Kiviaho *et al.* used 10× Visium spatial transcriptomics data to successfully identify a subclone of club-like cells closely associated with resistance in CRPC^[173]^. They found that this cell population highly expressed genes such as polymeric immunoglobulin receptor (*PIGR*), secretoglobin family 3A member 1 (*SCGB3A1*), mucin 1 (*MUC1*), lipocalin-2 (*LCN2*), *CXCL1/2/8*, and *CCL20*, significantly activating the IL6–Janus kinase (JAK)–STAT3, TNF–NF-κB, p53–SASP, the inflammatory response, and chemokine signaling pathways^[173]^. As a result, these cells recruited and activated polymorphonuclear myeloid-derived suppressor cells, which expressed arginase-1 (ARG1), calprotectin (S100A8/A9), CXCR2, IL1B, IL10, TNF-alpha-induced protein 6 (TNFAIP6), and other molecules, creating an immunosuppressive microenvironment^[173]^. Similarly, Mori *et al.* jointly employed 10× Visium spatial transcriptomics and snRNA-seq to identify a unique drug-resistant site in OCCC, which was formed by a group of resistance-associated tumor cells and CAFs^[46]^. They further revealed a positive feedback signaling circuit within this site involving tumor cells and CAFs, which ultimately led to treatment failure with carboplatin^[46]^.

Romero *et al.* applied 10× Visium spatial transcriptomics combined with snRNA-seq to prostate cancer to study the lineage transdifferentiation from prostate adenocarcinoma (PRAD) to androgen receptor signaling inhibitor (ARSI)-resistant neuroendocrine prostate cancer (NEPC)^[174]^. Their research revealed that the achaete-scute homolog 1 (ASCL1)-positive NEPC cell population originated from a neuroendocrine cell population within KRT8^+^ luminal progenitor cells, which were shown to be spatially dependent on the TME, thereby providing a potential spatial marker for therapeutic resistance^[174]^. Rubinstein *et al.* utilized 10× Visium spatial transcriptomics to describe the spatiotemporal heterogeneity of tumor tissue across different stages of dabrafenib plus trametinib combination therapy for BRAF V600E-mutated melanoma^[175]^. The study revealed remarkable spatiotemporal tumor heterogeneity during treatment. Spatially, a center-to-periphery pathway gradient was observed, with drug-resistant lineages located closer to the tumor margin^[175]^. Temporally, programmed transcriptional alterations emerged early during treatment, including upregulation of aerobic oxidative phosphorylation and invasiveness, with concomitant cell-cycle suppression, and persisted stably in MRD, potentially contributing to chemotherapy resistance^[175]^.

Agostini *et al.* employed GeoMx digital spatial profiling (DSP) whole-transcriptome analysis to study IPMN, the precursor lesion of PDAC, and identified an expression gradient of the mucin O-glycosylation pathway from low-grade to high-grade IPMN^[43]^. High-grade IPMN secreted increased O-glycosylated mucins with significantly upregulated glucosaminyl (N-acetyl) transferase 3 (GCNT3), thereby shaping a unique immunoevasive microenvironment that drove gemcitabine and nab-paclitaxel chemotherapy resistance^[43]^. Shiau *et al.* analyzed the mechanisms of resistance to neoadjuvant chemotherapy [8-12 cycles of FOLFIRINOX followed by fractionated radiotherapy (30-50 Gy equivalent dose in 2 Gy fractions) concurrent with 5-FU or capecitabine] in PDAC samples using CosMx spatial molecular imager (SMI) single-cell spatial transcriptomics^[111]^. Based on these data, the authors developed spatially constrained optimal transport interaction analysis (SCOTIA), an optimal transport model featuring a cost function that incorporates spatial distance and ligand-receptor gene expression, which revealed significant alterations in ligand-receptor interactions between CAFs and malignant cells under therapeutic stress^[111]^. The analysis confirmed that pathways associated with chemokines, cytokines, matrix remodeling, and immune regulation were significantly enhanced^[111]^. Notably, signaling within the IL-6 family, e.g., cardiotrophin-like cytokine factor 1 (CLCF1)–ciliary neurotrophic factor receptor (CNTFR) and leukemia inhibitory factor (LIF)–IL-6 cytokine family signal transducer (IL6ST), was enriched in the CAF-to-cancer-cell direction and was accompanied by JAK/STAT activation, ultimately conferring chemotherapy resistance to tumor cells^[111]^.

#### Spatial epigenomics

Spatial epigenomics is a major application of spatial omics technologies that enables analysis of the spatial organization of gene expression and associated epigenetic modifications across tissues at varying resolutions^[91]^. As previously mentioned, detecting epigenetic modifications at single-cell level plays a crucial role in the identification of tumor resistance mechanisms and the development of therapeutic approaches. Spatially resolving epigenomics *in situ* within tissue sections not only provides high resolution but also yields spatial distribution information. Additionally, combining multiple tissue sections can fully reveal the spatiotemporal tumor heterogeneity caused by epigenetic modifications. A growing number of technologies have recently emerged to enable spatially resolved analysis of epigenetic modifications. Although these approaches have not yet been applied to the study of tumor drug resistance, exploring spatial epigenetic heterogeneity may provide valuable insights for future research in this area^[55]^.

#### Spatial proteomics

While the analysis of protein expression and spatial distribution using traditional staining and immunofluorescence has long been a central approach for characterizing tissue architecture, technical limitations such as spectral overlap have constrained high-throughput analyses of protein localization^[91]^. Recently developed technologies are capable of simultaneously detecting multiple proteins, enabling spatial proteomics detection and analysis.

Kulasinghe *et al.* performed proteomic analysis of TNBC tissues treated with adjuvant chemotherapy comprising 5-FU, epirubicin, and cyclophosphamide using Nanostring GeoMx DSP^[176]^. The authors identified numerous protein markers associated with tumor response to chemotherapy and their underlying mechanisms. In tumors, granzyme A (GZMA), stimulator of interferon response cGAMP interactor (STING), and fibronectin were positively correlated with chemotherapy response, whereas CD80 was negatively correlated. In the tumor stroma, estrogen receptor alpha (ER-α) expression was positively correlated with chemotherapy response, whereas 4-1BB and melanoma antigen recognized by T-cells-1 (MART1) were negatively correlated. Similarly, Li *et al.* utilized RNA-seq combined with fluorescence *in situ* hybridization (FISH)/immune histochemistry (IHC) and DSP in a Phase II neoadjuvant clinical trial for HER2-positive breast cancer to characterize treatment resistance arising from HER2 and Erb-B2 receptor tyrosine kinase 2 (ERBB2) expression heterogeneity within tumor tissues^[177]^. Spatial-CITE-seq extends the CITE-seq technology to individual spatial spots within tissue sections. Although it has not yet been applied to drug resistance-related research, this technology holds considerable promise in such studies^[118,178]^.

Bouchard *et al.* analyzed the spatial heterogeneity in LUAD treated with erlotinib using spatial multiplexed immunofluorescence on the PhenoCycler. This study revealed that CAF-mediated spatial reorganization and tumor–stroma alignment could persist or regenerate under EGFR inhibition and were correlated with drug resistance^[179]^. Jhaveri *et al.* analyzed the spatial proteomics of HNSCC tissues treated with pembrolizumab using co-detection by indexing (CODEX) with a 101-antibody panel^[180]^. The study identified four metabolically distinct tumor regions and six spatially distinct neighborhoods enriched in different immune subpopulations. It was also found that M2 macrophages localized closer to tumor cells in glucose-6-phosphate dehydrogenase (G6PD)- and MMP9-overexpressing regions, where they exhibited immunosuppressive activity, providing new insights into TME features associated with response and sensitivity to ICI therapy^[180]^.

A study by Fan *et al.* employed laser capture microdissection (LCM)-MS-based spatial proteomics together with scRNA-seq to analyze cervical squamous cell carcinoma (CSCC) with poor response to ICI^[120]^. They identified bidirectional interactions between epithelial-cytokeratin tumor state malignant epithelial cells and mannose-6-phosphate (MP6) positive and MMP11^+^ CAFs in key non-responsive regions, which formed an immune-rejecting microenvironment through FABP5-mediated TGF-β signaling^[120]^. Spatially, MP6 co-localized with immune-depleted and CAF-enriched regions, while TGF-β activity showed peripheral ring-like enrichment along MP6 margins, thus defining a unique resistance niche^[120]^.

#### Spatial metabolomics

Spatial metabolomics supports the characterization of histological and functional properties of intact tissue sections and facilitates comprehensive analysis of the metabolic profiles of tumor cells and stromal regions^[181]^. In HER2-positive advanced gastric cancer treated with trastuzumab, Wang *et al.* analyzed the spatial metabolome of pre-treatment biopsy samples from 42 patients using matrix-assisted laser desorption/ionization and Fourier transform ion cyclotron resonance imaging MS (MALDI-FT-ICR IMS) and identified extensive metabolic heterogeneity within tumor tissues^[182]^. Quantification based on Simpson’s diversity index revealed a significant correlation between high metabolic heterogeneity and increased sensitivity to trastuzumab^[182]^. Clustering based on metabolic status showed that multiple pathways, including nucleotide, carbohydrate, and amino acid metabolism, were collectively downregulated in the subclusters most strongly associated with drug resistance^[182]^. Conversely, carbohydrate and amino acid metabolism were upregulated in the subclusters exhibiting the highest sensitivity to trastuzumab^[182]^. In NSCLC, Shen *et al.* analyzed the metabolic status of platinum-resistant samples following neoadjuvant chemotherapy using MALDI-FT-ICR MS^[183]^. They found that elevated levels of PC typically correlated with energy enrichment, enhanced proliferation, and poor prognosis in tumor regions, whereas in the stroma, glycerophospholipid metabolism exerted the strongest influence on prognosis and chemotherapy response^[183]^. Although this study did not deeply explore the mechanisms linking altered metabolic states to drug resistance, the tumor and stroma classifiers developed based on metabolic signatures still presented high accuracy and practical value in patient risk prediction^[183]^.

Ji *et al.* utilized spatial metabolomics coupled with spatial transcriptomics to analyze key metabolic differences between treatment-sensitive and treatment-resistant samples from nasopharyngeal carcinoma (NPC) patients receiving cisplatin-based chemoradiotherapy and PD-1 immunotherapy^[184]^. The authors observed elevated activity in branched-chain amino acid, glutamine, and fatty acid metabolism in treatment-sensitive samples, whereas the same metabolic pathways exhibited significantly reduced activity in treatment-resistant samples^[184]^. Additionally, the authors identified spatial microdomains within the same NPC sample (tumor, fibroblast, immune, tumor-immune mixed, and normal epithelial zones) and significant variations in branched-chain amino acid-, glutamine-, and lipid-related metabolites across regions^[184]^. Moreover, the spatial distribution patterns of metabolites differed between treatment-sensitive and treatment-resistant patients within these zones, suggesting widespread metabolic heterogeneity within tumors that directly correlated with treatment sensitivity^[184]^.

## PERSONALIZED THERAPEUTIC STRATEGIES TO OVERCOME TUMOR DRUG RESISTANCE

**Figure 5:**
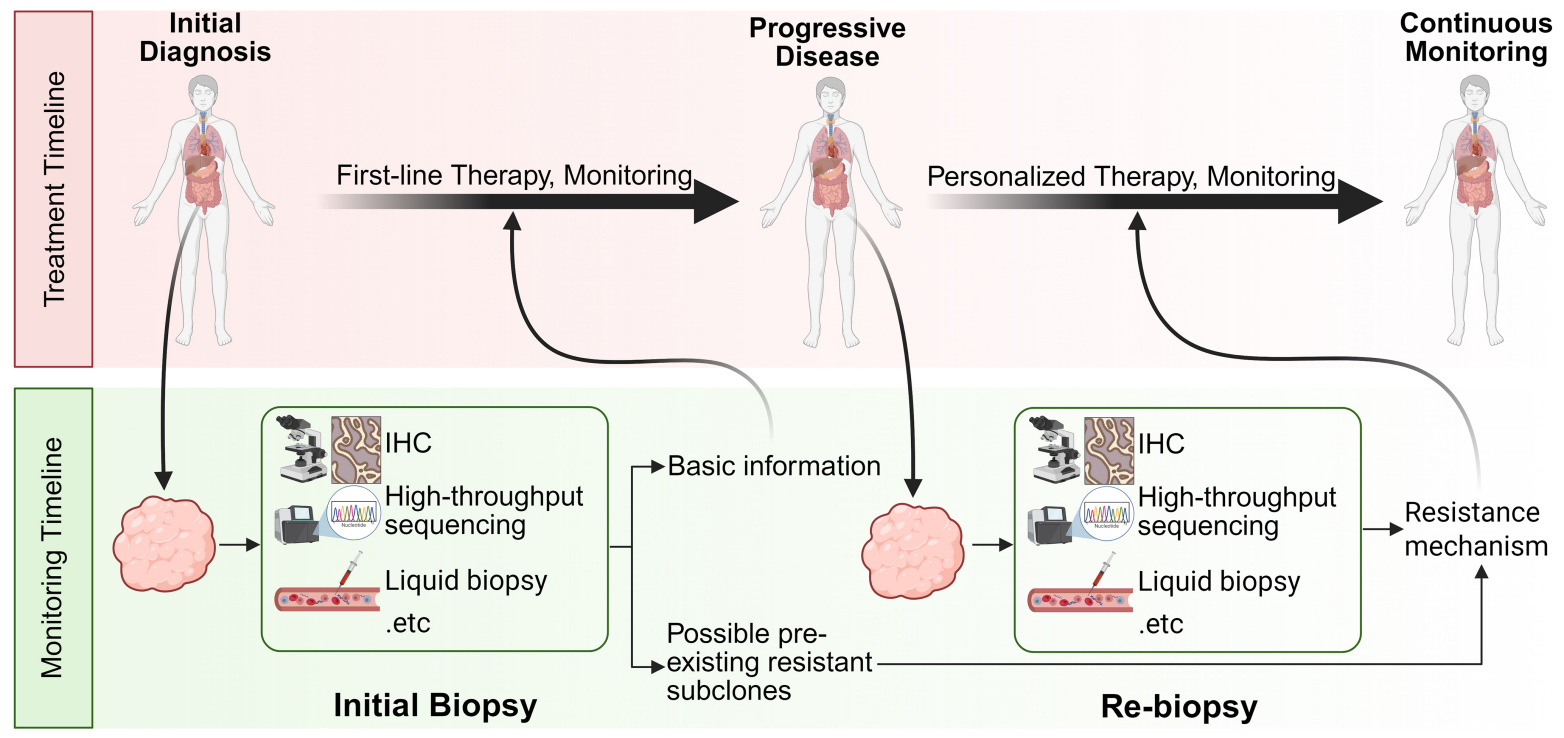
Flowchart of personalized cancer treatment guided by continuous monitoring to overcome drug resistance. For cancer patients who develop drug resistance during first-line treatments such as radiotherapy, chemotherapy, and targeted therapy, disease progression is continuously monitored and relevant research findings are integrated to precisely identify resistance mechanisms at an early stage. This approach supports the timely development and implementation of personalized therapeutic strategies. Created in BioRender. Da, E. (2026) https://BioRender.com/cg78gsu. IHC: Immunohistochemistry.

Single-cell and spatial omics technologies provide important insights into the mechanisms underlying tumor drug resistance, but these insights alone are not sufficient. The clinical value of characterizing resistance landscapes lies in their ability to inform treatment decisions. To effectively translate multi-omics findings into clinical benefits, personalized therapeutic strategies are needed that incorporate dynamic, biology-driven clinical decision-making to address the continuous generation of resistant clones [Figure 5]. In clinical practice, tumor management may benefit from shifting from one-time assessment to a continuous monitoring approach. At the time of clinical relapse, re-biopsy can help identify adaptive resistance mechanisms that have emerged during treatment. This information can guide the timely personalization of therapeutic strategies, such as rationally designed combination therapies or adaptive sequential drug replacements, with the aim of controlling tumor evolution and restoring therapeutic efficacy.

### Combination therapy

As demonstrated in many of the cases discussed in previous sections, combination therapy can simultaneously target multiple pathways, effectively overcoming drug resistance caused by the failure of single-target treatments. Pediatric GBM often fails to respond to single-target CAR-T therapy due to the selective proliferation of tumor subclones with antigen loss or mismatch, followed by immune escape^[141]^. Tian *et al.* successfully suppressed subclonal selection and subsequent immunoescape under therapeutic pressure using a glypican 2 (GPC2)/CD276 bicistronic CAR (BiCisCAR) that presented enhanced antitumor activity, persistence, and anti-fatigue properties^[141]^. In a study by Li *et al.*, the resistance mechanism in Olaparib-resistant breast cancer was found to be highly correlated with Rps19-C5aR1 signaling between the tumor cell and a specific macrophage subclone, CAM_C3, in the drug-resistant microenvironment^[47]^. The authors combined the specific Rps19 antagonist PMX53 with the original Olaparib treatment regimen, successfully inhibiting CAM_C3 and restoring tumor tissue sensitivity to Olaparib, thereby reversing the resistance phenotype^[47]^.

Liu *et al*. also identified co-localization of SPP1^+^ macrophages and CAFs within the TIB structure during ICI treatment of HCC and found that the ligand-receptor pair signaling from SPP1^+^ macrophages to CAFs was crucial for establishing the immunosuppressive microenvironment^[44]^. Based on this information, they specifically blocked the macrophage SPP1 axis using a neutralizing monoclonal antibody, successfully disrupting the TIB structure and increasing CD8^+^ T cell infiltration while decreasing CAF infiltration. This approach showed significant synergistic effects when combined with anti-PD-1 therapy^[44]^.

The efficacy of chemotherapy or immunotherapy for PDAC is often suboptimal, potentially due to a highly fibrotic and immunosuppressive TME^[185]^. These resistance niches feature dense stroma, poor vascularization, and elevated interstitial pressure, forming “cold” tumors that restrict immune cell infiltration and activation, and consequently conferring resistance to standard chemotherapy or ICIs^[185]^. Conversely, Carbone *et al.* demonstrated that L19-IL2 fusion protein therapy spatially coordinated the massive influx of cytotoxic CD8^+^ T cells and NK cells into the tumor core^[185]^. Thus, it effectively transformed the “cold” TME into a “hot” TME, potentially reducing resistance to conventional cancer therapies. This approach delivered favorable therapeutic outcomes both as monotherapy and in combination with FOLFOX^[185]^. Zhang *et al.* used Stereo-seq spatial transcriptomics technology combined with snRNA-seq on four cases of primary ER^+^/HER2^-^ breast cancer and found that during endocrine therapy resistance, the abundance of tumor-infiltrating T cells decreased^[186]^. Further analysis revealed that the cGAS [cyclic guanosine monophosphate–adenosine monophosphate (GMP-AMP) synthase]–STING (stimulator of interferon genes) pathway was blunted in endocrine-resistant cells, leading to enhanced AKT1 phosphorylation, which bound to the kinase domain of TANK-binding kinase (TBK1) through its own kinase domain, thereby blocking the formation of the STING/TBK1/interferon regulatory factor 3 (IRF3) ternary complex and inhibiting downstream STING signaling^[186]^. This positive feedback loop established the endocrine resistance mechanism in drug-resistant tumors^[186]^. Based on this, the authors proposed and demonstrated that combined treatment with a STING agonist and an AKT1 inhibitor successfully reversed endocrine resistance, offering a promising approach to overcome endocrine resistance in estrogen receptor (ER)-positive (ER^+^)/HER2- breast cancer^[186]^.

Despite the enormous potential of combination therapies in overcoming tumor heterogeneity and resistance, a major clinical challenge must be addressed: the amplified risk of cumulative toxicities and adverse side effects compared to single-agent treatments^[187]^. Therefore, accurately distinguishing pharmacological effects on malignant subclones from those on healthy cells is critical when designing personalized combinatorial regimens. In this context, single-cell and spatial multi-omics technologies present a critical advantage. By simultaneously profiling resistant tumor environments and adjacent normal tissues at high resolution, these tools allow detailed comparison of the baseline molecular profiles of tumors and healthy cells. This information enables precise identification of therapeutic targets and specific intercellular communication networks within drug-resistant TMEs that are largely absent from normal tissues. For example, in gastric cancer, simultaneously considering the spatial distribution of HER2 and the activation patterns of downstream signaling pathways can effectively guide the intra-tumoral injection of trastuzumab-loaded nanoparticles into HER2-enriched regions, thereby enhancing therapeutic efficacy^[188]^. Furthermore, drug-resistant targets often exhibit significant heterogeneity compared to normal tissues. Combination therapies directed against such targets to overcome resistance can minimize adverse effects. For instance, in the treatment regimen combining savolitinib with osimertinib for osimertinib-resistant NSCLC caused by MET amplification, both the Phase II clinical study SAVANNAH^[189]^ and the Phase III clinical study SACHI^[190]^ indicated that this regimen can reverse MET amplification-related resistance while effectively ensuring treatment safety. This high-definition multi-omics approach can expand the therapeutic window by informing the rational design of combination strategies that maximize targeted cytotoxicity against resistant clones while minimizing collateral damage to healthy organs.

### Drug replacement and staged therapy

Drug resistance arising from tumor heterogeneity requires a period of evolutionary or acquired mutation stabilization before manifesting in the tumor’s bulk phenotype. By understanding the cyclic progression of resistance acquisition and periodically adjusting treatment strategies, the formation of stable resistance within the tumor can be prevented, thereby maintaining the drug’s cytotoxic efficacy.

Jerby-Arnon *et al.* identified an immune-resistant tumor cell state in ICI-resistant melanoma that which was associated with an immune-resistance program driven by the CDK4/6–E2F–MYC axis^[132]^. Based on this finding, abemaciclib (a CDK4/6 inhibitor) was considered as an immune re-sensitizer^[132]^. After comparing ICI monotherapy, abemaciclib monotherapy, ICI plus abemaciclib combination therapy, and ICI plus abemaciclib sequential therapy, the authors found that the two-step approach - first administering ICI alone followed by ICI plus abemaciclib combination therapy - offered unique advantages in treatment potential^[132]^. The following mechanism could explain that: (1) ICI first targets CD8^+^ T cells in the tumor periphery, activating immune cells while simultaneously inducing tumor cells into an immune-resistant state; (2) abemaciclib inhibits the CDK4/6–E2F–MYC resistance pathway, thereby restoring tumor cell immunosensitivity^[132]^. This allows the tumor cells to be attacked by the pre-activated CD8^+^ T cells, achieving optimal antitumor activity. In contrast, simultaneous administration only suppresses tumors in the short term, but the lack of sustained immune response prevents long-term efficacy^[132]^.

In the early stages of TNBC, EZH2 inhibitors may have induced resistance by promoting reduced H3K27me3 levels. However, in patients who have already developed resistance to 5-FU and capecitabine, these inhibitors could instead suppress resistance and enhance treatment efficacy by regulating H3K27me3 levels. As a result, flexibly adjusting the timing and combination of EZH2 inhibitors with 5-FU and capecitabine according to each patient’s resistance status may represent an effective strategy for overcoming resistance^[27,191]^. In CLL cases where selective subclone amplification led to ibrutinib resistance, Cadot *et al.* demonstrated that leukemic cells remained sensitive to venetoclax despite developing resistance to ibrutinib^[34]^. *In vitro* experiments revealed significant disease suppression after switching to venetoclax^[34]^.

Despite the theoretical advantages, implementing staged or adaptive therapies in standard clinical care faces significant practical barriers. Clinicians must navigate the risk of rapid disease flare during drug holidays and manage cumulative or overlapping toxicities from sequential regimens. Furthermore, the success of staged therapy heavily relies on determining the precise timing for treatment switches, which is currently hindered by the lack of standardized, high-frequency, and non-invasive monitoring tools in routine practice.

### Developing novel therapeutic strategies for overcoming drug resistance

**Figure 6:**
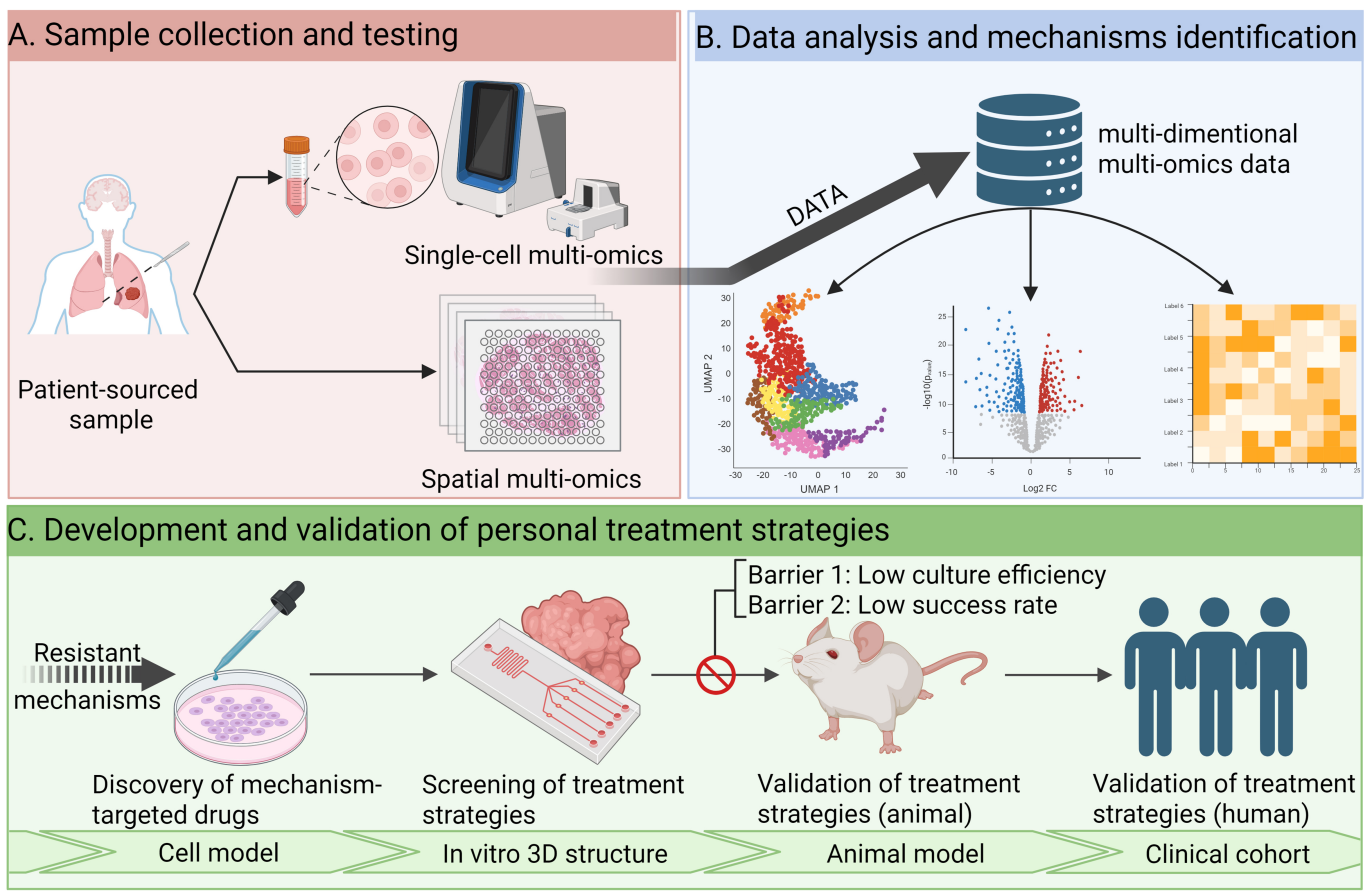
Development of personalized treatment strategies for drug resistance. (A) High-resolution single-cell and spatial multi-omics analyses of patient samples to intratumoral heterogeneity characterization; (B) Computational analysis and bioinformatics tools to accurately identify drug-resistant subclones, decode subclone-specific characteristics, cell-cell interactions, and bypass signaling pathways, thereby computationally identifying potential drug resistance mechanisms and therapeutic targets; (C) Sequential use of cell models, 3D *in vitro* models, and animal models for identification of resistance mechanisms related targets, selection and validation of the most appropriate and effective drug types and personalized treatment strategies, and ultimately validation of their efficacy in clinical trials. The main challenges currently lie in reproducing *in vivo* the therapeutic effects of *in vitro* models and the low efficiency of constructing these models. Created in BioRender. Da, E. (2026) https://BioRender.com/ys10nrf.

As tumor heterogeneity often leads to the coexistence of multiple resistant subpopulations within the same patient or tumor mass, developing comprehensive treatment strategies is challenging^[35,36]^. Single-cell and spatial multi-omics tools enable detailed characterization of tumor drug-resistant subclones in patients, and support workflows ranging from the identification of resistance mechanisms to the development of personalized therapeutic strategies. The development of novel personalized treatment strategies typically involves several stages [Figure 6], including sample collection and testing, data analysis and identification of drug resistance mechanisms, and the formulation of treatment protocols followed by feasibility studies. Among these stages, the screening and the validation of treatment strategies are the most critical ones. As mentioned earlier, single-cell and spatial multi-omics technologies have revealed the complex internal structure of tumor tissues with unprecedented molecular resolution. The integration and analysis of multidimensional data have enabled the identification of mechanisms of drug resistance. However, a key challenge in developing personalized treatment strategies remains the identification of appropriate drug types and combinations, and the effective translation of insights gained from resistance mechanisms into actionable therapeutic strategies.

The coexistence of multiple drug-resistant subclones poses significant challenges for treatment of drug-resistant tumors. Only by fully considering each individual subclone can a comprehensive treatment plan be developed. For example, NSCLC patients carrying activating EGFR mutations often develop resistant subclones with secondary EGFR mutations, as well as subclones that rely on alternative mechanisms like MET gene amplification for survival following EGFR inhibitor treatment^[33,192]^. Different drug-resistant subclones exhibit different drug sensitivities and survival capabilities^[192,193]^. In this context of concurrent multidrug resistance, neither EGFR-TKI targeted against mutations nor MET inhibitors alone can induce more than transient tumor regression^[194]^. This is because the uninhibited subclones will rapidly unleash their growth potential and lead to recurrence. A combined treatment strategy targeting different drug-sensitive subclones may be a key approach to improving patient prognosis^[33,195]^.

Traditional two-dimensional cell culture struggles to replicate the complexity of the TME, while extensive animal testing raises ethical concerns. The development of effective therapies against complex TMEs, such as coexisting multi-tumor subclones, requires the simulation of complex tumor tissues *in vitro*. Patient-derived organoids (PDOs) generated by isolating and culturing cells from patient samples can effectively replicate the physiological conditions of organs or tissues, providing a more accurate model for studying tumor biology and evaluating therapeutic responses compared to traditional two-dimensional cell cultures^[196,197]^. For example, Um *et al.* successfully utilized HNSCC PDOs to simulate tumor evolution under cisplatin treatment pressure and identified key drug-resistant subclones^[198]^. Using PDOs, the study by Vlachogiannis *et al.* found a good correlation with clinical responses in chemotherapy-treated patients during drug sensitivity testing, indicating that PDOs can effectively replicate the composition and behavior of tumors^[199,200]^. In a study involving 54 patients, Beutel *et al.* found that PDOs enabled the development of personalized combination drug regimens within a shorter timeframe (median 53 days) and facilitated predictive scoring, highlighting the significant potential of PDOs for informing the development of treatment strategies in clinical practice^[201]^. Tumor assembloids technology, achieved by integrating organoid technology with droplet microfluidics, goes a step further. This technology more effectively captures tumor-to-tumor and intra-tumor heterogeneity, genomic and transcriptomic landscapes, and TME cellular diversity^[202]^. In lung cancer-derived assembloids, Zhang *et al.* successfully replicated the heterogeneity of drug resistance arising from CAF heterogeneity and variations in the spatial distance between tumor cells and CAFs, demonstrating the immense potential of assembloids for studying tumor heterogeneity and developing targeted therapeutic strategies^[202]^. Although the approach of using PDO models to simulate the complex composition of *in vivo* tumors for *in vitro* drug screening has not demonstrated significant efficacy in clinical trials and suffers from drawbacks such as low culture success rates and excessive time consumption^[203]^, comprehensively analyzing tumor composition and understanding intrinsic heterogeneity remains highly valuable for cancer treatment^[204]^. In the future, technologies such as high-throughput automated microfluidic culture and organ-on-a-chip systems may help address the high failure rates and low culture efficiency observed in workflows spanning *in vitro* 3D structure screening, animal studies and clinical cohort validation^[205-207]^. These advances may improve the development of personalized treatment strategies for diverse forms of tumor drug resistance and help shorten the development timelines. In parallel, the refinement of *in vitro* models may enhance the reproducibility of *in vitro* findings in *in vivo* settings. Integrating high-throughput drug screening strategies using *in vitro* 3D models with subsequent validation in animal studies and clinical cohorts offers an approach that improves efficiency while reducing reliance on animal experimentation, supporting more effective and resource-efficient cancer therapy development. Preclinical *in vitro* models provide valuable functional validation for the screening and development of therapeutic approaches, but their ultimate translational impact depends on large-scale clinical trials that employ heterogeneous testing strategies. Landmark clinical programs, such as TRACERx or SAVANNAH and SACHI that verify new treatment strategies in a clinical cohort study, have significantly shaped our understanding of how intra-tumoral heterogeneity and subclonal evolutionary dynamics dictate clinical outcomes and resistance in real time. These ongoing clinical efforts play an important role in linking omics-guided subclone identification and resistance mechanisms analysis with the development of personalized therapeutic strategies and improvements in overall survival.

## CONCLUSION

Tumor heterogeneity is the main driver of drug resistance, manifesting across both spatial and temporal evolutionary dimensions. In this review, we systematically summarize how pre-existing resistant clones, adaptive reprogramming, and diverse TMEs collectively contribute to drug resistance, along with common mechanisms underlying failure of targeted therapies.

Addressing this complexity using single-cell and spatial multi-omics technologies is not merely an academic exercise but a clinical imperative. Overcoming drug resistance requires tailored therapeutic strategies, including rationally designed combination therapies and adaptive staged treatment approaches. While clinical implementation of single-cell and spatial multi-omics approaches remains limited, several low-cost and rapid-turnaround diagnostic methods - such as multiplex immunohistochemistry, targeted next-generation sequencing panels, and liquid biopsy-based detection of circulating tumor DNA (ctDNA) carrying resistant clonal mutations - have facilitated the translation of laboratory findings into clinical practice^[208-215]^. Application of these approaches in clinical settings may enable the identification of existing resistance mechanisms or the anticipation of emerging resistance, thereby supporting more informed treatment decisions.

Looking ahead, a key opportunity in clinical research lies in establishing a “discovery-to-clinical-translation” pipeline. Integrating high-resolution single-cell and spatial multi-omics data with advanced computational approaches will empower clinicians to precisely decode tumor-stroma interactions and monitor clonal evolution. Furthermore, coupling these multi-omics analyses with functional *in vitro* validation platforms represents a transformative research direction. Together, these approaches may support a shift in personalized oncology from static, single-target strategies toward more dynamic, multi-targeted, and functionally informed treatment strategies. Ultimately, by preemptively targeting subclonal vulnerabilities, such strategies have the potential to improve management of heterogeneous resistance, reduce MRD, and extend patient survival.

## DECLARATIONS

### AI and AI-assisted tools statement

During the preparation of this manuscript, the AI tool Gemini (version 3 Pro, released 2025-11-18) was used solely for language editing. The tool did not influence the study design, data collection, analysis, interpretation, or the scientific content of the work. All authors take full responsibility for the accuracy, integrity, and final content of the manuscript.

### Copyright

© The Author(s) 2026.
